# Parental Loss and Mental Health in Post-Khmer-Rouge Cambodia

**DOI:** 10.1007/s11113-024-09894-3

**Published:** 2024-06-25

**Authors:** Patrick Heuveline, Angela K. Clague

**Affiliations:** 1 California Center for Population Research (CCPR) at the University of California, Los Angeles (UCLA), Los Angeles, USA; 2 The RAND Corporation, Santa Monica, USA

**Keywords:** PTSD, Mental Health, Orphan, Cambodia

## Abstract

Adverse childhood events (ACE) may have lasting consequences throughout the life course. We focus on one particular type of ACE, parental loss in Cambodia—a country that lost nearly 25% of its population during the 1975–79 Khmer-Rouge regime—and on mental health disorders, one of the potential mechanisms through which ACE may have long-term consequences. Self-reports of symptoms that map on to the Diagnostic and Statistical Manual of Mental Health Disorders (DSM) criteria for anxiety, depression, and post-traumatic stress disorder (PTSD) were collected from 4,405 adults aged 20 and over. We first assess exposure to traumatic events and the prevalence of anxiety, depression, and PTSD using the DSM and alternative criteria. Based on the DSM criteria and previously validated Likert-scale thresholds, we find a high prevalence of anxiety (56.0%), depression (42.8%), and PTSD (2.3%), and even higher levels even among KRR survivors. We then use logit models to analyze the effect of parental loss before age 20 on the likelihood of having experienced traumatic events and experiencing mental health disorders. We find the loss of one parent increases the likelihood of full-PTSD symptoms, but the loss of both parents does not. These findings may result from positive selection into better-off households for orphans whose parents have both died but may also reflect the grief-related difficulties faced by the surviving parent of paternal or maternal orphans. While alternative thresholds for PTSD produced higher prevalence estimates, these measures did not perform better for assessing the effect of parental loss on mental health.

## Introduction

Adverse childhood events (ACE) may have lasting consequences throughout the life course, possibly affecting physical and mental health, employment, and marital stability (Jones et al., 2018). In this paper, we focus on parental loss, which can be expected to be among the most impactful of these ACE. Establishing causality can be difficult, however, due to processes that begin before the loss of a parent and mitigating circumstances thereafter. In most countries, early parental death has become rare, fortunately, but more selective as a result, since parents who pass away in low-mortality settings can be expected to belong to households that were already socio-economically disadvantaged (negative selection). Parental death can also be followed by adaptive processes, such as fosterage, aimed at alleviating some of the effects of parental loss on orphans, thus complicating the identification of direct linkages between parental death and consequences later in life. These processes may differ for paternal and maternal orphans, and for orphans who lost both parents (bilateral orphans thereafter). For bilateral orphans in particular, parental loss can be expected to lead to a household transition. Households with more resources might be more likely to take orphans in (positive selection). These processes have been studied mostly in the context of HIV/AIDS epidemics in sub-Saharan Africa, where bilateral orphans were found to live in better-off households than non-orphans (Bicego et al., 2003) or than paternal orphans (Case et al., 2004).

Our study is set in Cambodia. During the 1975–79 Khmer Rouge regime (KRR), the country lost nearly 25% of its entire population, with particularly high mortality among adult males (Heuveline, 2015). This implies that an unusually large share of today’s adult population has lost a parent, or both, at an early age. These tragic circumstances provide a unique context to study the diversity of experience across maternal, paternal, and bilateral orphans. While there were targeted executions following the KRR takeover, the general deterioration of living conditions during this period caused mortality to surge as nearly the entire population experienced deficits between caloric needs and the intake from the food rations the government handed down to the population, increased vulnerability to infectious diseases, and violent, possibly fatal punishment for even minor rule violations. Mortality varied across the country but, because people were assigned to live in specific locations, geographic mobility to areas with slightly better living conditions was not possible. The KRR also abolished money, and the only use of prior economic advantage was limited to bartering valuables, which was also punishable and saved for rare occasions rather than daily needs. In this context, excess adult mortality was widespread, and we expect negative selection into orphanhood to be less of an issue than in most other settings.

With respect to the long-term consequences of parental loss, we focus here on mental health disorders, one of the potential mechanisms through which parental loss may affect other outcomes later in life. We first document exposure to traumatic events, and the prevalence of anxiety, depression, and somatic symptoms overall and among KRR survivors in particular. We then focus on post-traumatic symptom disorder (PTSD) which most clearly links mental health symptoms to exposure to traumatic events earlier in life. We again document the prevalence of PTSD in the general population and among KRR survivors. Last, we analyze whether, net of exposure to traumatic events, mental health disorders are more common among adults who have lost a parent.

## Literature Review

A definition of post-traumatic symptom disorder (PTSD) was first included in the 3rd edition of the American Psychiatric Association Diagnostic and Statistical Manual of Mental Health Disorders (DSM) in 1980. Alongside depression, elevated rates of PTSD have since been documented in populations exposed to mass conflict and displacement (Steel et al., 2009). The population of Cambodian refugees who began to arrive in North America beginning in the early 1980s is no exception.

In a random sample of households from a Cambodian community in Long Beach, California, 99% of adults aged 35 to 75 years who lived in Cambodia during the KRR and immigrated to the United States prior to 1993 reported starvation during the regime and 90% had a family member or friend murdered. Among these survivors, 51% were diagnosed with depression and 62% with PTSD (Marshall et al., 2005). In an earlier study, as many as 86% of Cambodian refugees who had resettled to Greensboro, NC, were found to meet the DSM criteria for PTSD (Carlson & Rosser-Hogan, 1991). Elevated levels were also found among the children of refugees. In a sample of Cambodian youths and their mothers in Portland, Oregon, 12.9% were diagnosed with major depression disorder and, using the DSM criteria, 26.5% with PTSD (Sack et al., 1996).

Refugee populations are likely to be selected on traumatic exposure, but the high prevalence of mental health disorders has also been documented in the general population in Cambodia. A study conducted in 3 different geographic areas found 24.8% of KRR survivors aged 16 and older to suffer from PTSD (de Jong et al., 2001). In a household survey conducted in one province (Kampong Cham), symptoms meeting the DSM criteria for high anxiety were reported by 53% of adults aged 20 years or older, 42.4% of them for depression, and 7.3% of them for PTSD (Dubois et al., 2004). In a 2006–2007 national probability sample survey, 11.2% of all respondents, and 14.2% of those aged three and older at the time of the KRR, were assessed as probably suffering from PTSD (Sonis et al., 2009). To date the largest-scale investigation of mental health in Cambodia, a 2012 survey interviewed a randomized sample of 2,690 adults from 9 of Cambodia’s 25 provinces (Schunert et al. und.). The authors report that 92.3% of KRR survivors (those born in 1975 or earlier) have experienced at least one traumatic event, but so have 70.2% of respondents in later birth cohorts. Overall, 27.4% of all respondents were found to suffer from probable anxiety, 16.6% from probable depressive disorders and 2.7% from probable PTSD. Prevalence levels were higher among KRR survivors: 30.8% for anxiety, 18.6% for depression and 3.3% for PTSD.

Prevalence estimates in this and other large population-based surveys are not based on actual diagnostic interviews but on Likert-scale thresholds and self-reports regarding symptoms that map on to the DSM criteria for various disorders. While the survey descriptions of these symptoms and the thresholds for determining whether a respondent may present a mental health disorder have been extensively validated at the population level, the term “probable” disorder remains *de rigueur* to emphasize that these scales do not provide individual diagnostics. The Hopkins Scale Checklist (HSCL-25), for instance, is a 25-item list of symptoms that map on to the DSM criteria for anxiety, depression, and somatic symptoms. The Harvard Trauma Questionnaire (HTQ) is a two-part questionnaire including, first, a list of different traumatic events and, second, a list of symptoms that map on to the DSM criteria for PTSD. Both the HSCL-25 (Mollica et al., 1987) and the HTQ (Mollica et al., 1992) have been adapted to better capture the experience of Southeast-Asian refugees and translated into several languages including Khmer, the national language of Cambodia. The Khmer version of the HTQ includes a 41-item list of traumatic events and a 40-item list of PTSD symptoms. The first 17 of these symptoms are the original symptoms shown to map on to the 4th edition of the DSM (DSM-4) criteria for PTSD (Wilkins et al., 2011). The remaining symptoms were added specifically for Southeast-Asian refugees.

Studies reporting that the types of symptoms found among Cambodian refugees are very similar to those observed among survivors of traumatic experiences else-where notwithstanding (Carlson & Rosser-Hogan, 1993, 1994; Sack et al., 1997; Palmieri et al., 2007), the application of Western categories to diagnose mental health disorders has been criticized for lack of nuance or attention to context. Critiques are two folds. On the one hand, while they may seem to correspond to the DSM criteria for PTSD, some of the subjective experiences described by Cambodian refugees have been argued to belong instead to a normal rehabilitation process of “cultural bereavement” from traumatic experiences (Eisenbruch, 1991). On the other hand, several studies have identified idioms in Khmer that may express post-traumatic distress in a manner that would not necessarily be detected with the standard description of symptoms shown to map on to the DSM criteria for PTSD in Western contexts (Hinton et al., 2002, 2010; Chhim, 2013).

Furthermore, the death of a close family member is only included in the HTQ list of traumatic events when resulting from murder or unnatural causes. The above-mentioned general deterioration of living conditions, severe under-nutrition, and increased exposure to infectious diseases during the KRR implies that many of the KRR-era deaths, possibly the majority thereof, were not violent deaths (Sliwinski, 1995; Heuveline, 2001). While the incidence of major depressive disorder and PTSD has been found to be higher, and the trajectory of recovery to be slower after losses due to sudden and violent deaths than after those due to natural deaths (Schaal et al., 2010; Kristensen et al., 2012), the impact of bereavement also varies with the degree of closeness to the person lost (Heeke et al., 2017) and appears particularly severe following the loss of a parent during childhood or adolescence (Schaal et al., 2010; Morina et al., 2011). The HTQ list of traumatic events may not fully capture the traumatic experience of many who lost a parent, or both, at an early age during the KRR.

To our knowledge, the link between mental health disorders and orphanhood has not yet been studied in Cambodia. Ong et al.’s (2015) did find a high incidence of depressive symptoms among Cambodian orphans and vulnerable children aged 11 and older, which was associated with physical health and exposure to violence, but their study did not include a comparison group of non-orphans. In other armed-conflict settings, Hasanović et al.’s (2006) reports higher prevalence of depressive disorder and PTSD symptoms among children with a deceased parent in Bosnia and Herzegovina. In Rwanda, 44% of children living in child-headed households or in orphanages presented PTSD symptoms (Schaal et al., 2012). Several other studies in sub-Saharan Africa, where early parental loss is unfortunately less rare than in other parts of the world, report similar findings (Cluver & Orkin, 2009; Puffer et al., 2012).

## Data & Methods

All data used for this study are from *The Mekong Integrated Population-Registration Areas of Cambodia* project (MIPRAoC). The project combines a health and demographic surveillance system (HDSS) and occasional “rider” surveys. The HDSS covers seven population-registration areas (PRA thereafter). One of these PRA has been followed since 2000 as part of *The Mekong Island Population Laboratory* project (Inter-university Consortium for Political and Social Research, 2017). Added in 2008, the other six PRA were drawn across seven provinces from the Northern border with Laos to the Southern border with Vietnam, from a sample of roughly-equal-size areas within all the districts classified as rural in the 1998 General Population Census (GPC) and located along the Mekong River. MIPRAoC is thus designed to be representative of the population of these contiguous districts, where 20% of the rural households in the country resided at the time of the 1998 GPC.

At the time of the 2008 benchmark census, the resident population of the seven PRA was close to 60,000 (59,592 registered individuals). HDSS updates have been conducted biennially from 2010 to 2016. One of the selected PRA is located in Phnom Penh Province, and though classified as rural in the 1998 Census, has since been absorbed by the capital city’s urban agglomeration. In the process, its population has grown quite large, and only one third of this population has been retained in the biennial demographic updates and rider surveys. As a result, the size of the population followed in the HDSS updates since is slightly above 50,000.

A rider survey was conducted in connection with the 2012 and 2014 biennial updates (rounds 3 & 4). The rider survey addressed various dimensions of the residents’ welfare: mental and physical health, social relationships, and economic wellbeing. The sample included all the residents who were identified as maternal or bilateral orphans at round 1, a 50% random sample of those identified as paternal orphans at round 1, and a random sample of residents at the time of the survey (round 3 or 4). In our study, an orphan is defined as a person who has lost a biological parent by the age of 20 or younger. We use a cut-off age of 20 because of the role parents are expected to play not just during childhood development but also the transition to adulthood, including marriage arrangements (Heuveline & Kao Nakphong, 2023).

A total of 5,499 questionnaires were administered at round 3 or 4. After dropping a few round-3-and-4 duplicates (268), individuals who immigrated after round 1 (to ensure benchmark information was collected at the same point in time, 51), and individuals under the age of 20 at round 1 (to avoid censoring on exposure to orphanhood as defined herein, 775), the analytical sample consists of 4,405 adults aged 20 and over at the time of the 2008 benchmark census.

### Independent Variables

All independent variables are taken from the 2008 benchmark census. At the time of registration in the benchmark census, each household head provided for each household member their name (later replaced by a unique identifier), gender, birth date, relationship to the head, and parental information (is the mother/father alive; if so, where does s/he lives, else when did s/he die).

Our primary variable of interest is orphan status. As shown in Table 1, a little over 60% of the 4,405 respondents were orphans before age 20: 1,385 (32.5%) paternal orphans, 697 (16.4%) maternal orphans and 581 (13.1%) bilateral orphans.

Expecting those who experienced the 1975–79 KRR to have been exposed to more traumatic events and the effects of exposure to depend on the age at the time of exposure, we cluster birth cohorts as follows: 1960 and earlier (i.e., already 15 or older at the onset of the KRR; 28.5% of the respondents), 1961–1974 (i.e., under age 15 at the onset of the KRR, 42.6%), 1975–1978 (i.e., born during the KRR; 8.4%) and 1979 and later (i.e., born after the fall of the KRR, 20.5%). All models also control for sex (58.5% female), marital status (currently married, 81.5%; never married, 9.9%; separated or divorced, 3.3%; and widowed, 5.4%), and location, using 4 categories: (1) urban (the Province of Phnom Penh, the capital city, 29.6%), (2) peri-urban (rural districts nearly adjacent to Phnom Penh, 24.1%), (3) rural, close (rural districts relatively close to Phnom Penh, 29.9%) and (4) rural, remote (the rural districts in the PRA furthest from the capital city, 16.4%). Finally, we created a four-category variable for educational attainment: no formal schooling (20.6%), some primary-education schooling (48.8%), complete primary education only (18.7%), and complete lower secondary education (9.4%).

### Dependent Variables

All our dependent variables are taken from the rider survey administered during the 2012 and 2014 updates (Rounds 3 & 4). This rider survey consisted of several modules, one of which included a list of self-reports from the Khmer versions of the HSCL-25 and HTQ. Only reduced lists from the Khmer versions of the HSCL-25 and HTQ were fielded, however, as pilot testing raised two concerns: (1) in our study population, respondents frequently reported difficulty distinguishing between relatively similar items in these lists (e.g., difficulty concentrating v. difficulty paying attention), and (2) as again this module on mental health was only one of several modules in the rider survey, an excessive length of the survey overall could compromise the quality of the self-reports.

The self-reports that were collected only concerned 18 of the HSCL-25 symptoms, 17 of the 41 HTQ traumatic events, and 21 of the 40 HTQ symptoms (including all the 17 original symptoms based on DSM-4 criteria). From post-training discussion with interviewers, a summary self-rating of physical and mental status was also included with five possible answers: feeling (1) comfortable/in good health, (2) in pain/suffering, (3) indifferent, (4) happy, and (5) sad.

#### Traumatic Events

We created five dummy variables based on the 17 traumatic-event self-reports. The first variable captures whether the respondent personally experienced or was a direct witness of any traumatic event. The other four variables are based on a typology of traumatic events: deprivation, life-threatening situations, abuse, and the unnatural death of a friend, or family member, or any murder.

#### Anxiety, Depression, and Somatic Symptoms

We created three dummy variables based on the (two) anxiety, (seven) depression, and (nine) other somatic symptoms. As in the HSCL-25, respondents self-reported whether they experienced various symptoms using a scale from 1 (not at all) to 4 (very much). The two dummy variables for anxiety and depression were coded as 1 if on this scale self-reports for the corresponding symptoms averaged 1.75 or higher. The third dummy variable was coded as 1 if any of the nine other somatic symptoms was self-reported as experienced “quite a bit” or “very much”.

#### Post-Traumatic Stress

Respondents also self-reported experiencing PTSD symptoms using a scale from 1 (not at all) to 4 (very much). Each of the symptoms belong to one of three clusters identified in the DSM-4 definition: B (re-experiencing), C (avoidance) or D (increased arousal). Our first PTSD dummy variable follows the DSM-4 definition for probable PTSD which requires respondents to self-report experiencing “quite a bit” or “very much” at least 1 cluster-B symptom, 3 cluster-C symptoms and 2 cluster-D symptoms. We also created two variables corresponding to alternative criteria for “partial” PTSD, or “subsyndromal” PTSD, which has also been found to be associated with comorbid disorders (Dickstein et al., 2015). The “stringent” criteria for partial PTSD require 2 cluster-C symptoms (instead of 3 for full PTSD), combined, as in the criteria for full PTSD, with at least 1 cluster-B symptom and 2 cluster-D symptoms. The “lenient” criteria require 3 cluster-C symptoms or 2 cluster-D symptoms (not both), combined, as in the criteria for full PTSD, with at least 1 cluster-B symptom.

We also created three PTSD dummies based on the Likert-scale of self-reports irrespective of their clusters. The first two are coded as 1 if the scaled self-reports average 2.5 or higher. The only difference between these two dummy variables is that one only includes self-reports of the first 17 HTQ symptoms designed to map on to the DSM-4 criteria, whereas the second one includes self-reports of the 21 symptoms retained from the Khmer version of the HTQ questionnaire. The last dummy variable is coded as 1 if the respondent self-reports experiencing “quite a bit” or “very much” any of these 21 symptoms.

### Methods

Beyond the descriptive statistics, we explored the impact of parental loss before age 20 both on exposure to traumatic events and on mental health using logistic regression. Models predicting exposure to traumatic events include the independent variables described above except for educational attainment since traumatic exposure might have preceded the completion of formal education. The risk of traumatic exposure was assessed, first, with a single variable representing exposure to any trauma event and, second, using four variables representing exposure to the four types of traumatic events. Models predicting mental health outcomes include all independent variables, first without exposure to trauma events, then with exposure to any traumatic event and to one of the four types of traumatic events.

## Results

Exposure to traumatic events and the prevalence of various mental health disorders are shown in Table 2 for the different birth cohorts. The results confirm the very high rates of exposure to traumatic events in this population. Nearly three quarters of the respondents (73.7%) reported having directly experienced or witnessed at least one traumatic event. As expected, the proportion is higher among those born before the outset of KRR—with little difference between those under 15 (75.1%) and those 15 and older at the time (74.9%)—but is nearly as high among those born during the KRR (67.9%) or those born after (71.7%).

With respect to the different types of traumatic events, most frequently experienced or witnessed was deprivation (60.7% of the respondents, Table 2); next were life threatening situations (45.3.%), abuse (24.3%), and the unnatural death of a friend, or relative, or murder (15.1%). Experiencing or witnessing any of these types of events follows the same inter-cohort pattern as above: substantially higher proportions among pre-KRR birth cohorts, but far from negligible proportions among KRR and post-KRR cohorts. The gradient is steepest for unnatural death of a friend, or relative, or murder, which was directly experienced or witnessed by 11.8% of the respondents born during the KRR or after, compared to 16.3% of those born before the KRR and under age 15 at the outset and 16.7% of those age 15 and over at the outset.

Turning to self-rated status, the respondents born at the outset of KRR (50.3% of those under 15 and 38.2% of those 15 and older at the time) are much more likely to report being sad, suffering or in pain than those born during the KRR (27.1%) or those born after (23.6%). These proportions are slightly lower than the prevalence estimates for probable depression, which show the same inter-cohort pattern: a marked increase from 28.8% for those born after the KRR regime to 54.7% for those age 15 and over at the outset of the KRR. The prevalence of probable anxiety and other somatic symptoms are even higher than for depression. More than half of the respondents meet the criteria for probable anxiety (56.0%) and report at least one somatic symptom (57.4%). For both, the prevalence is highest for respondents who were age 15 and over at the outset of the KRR but, contrary to depression, is slightly higher for respondents born after rather than during the KRR (Table 2).

With respect to PTSD, applying DSM-4 criteria suggests that 2.3% of our respondents probably experience full PTSD, a prevalence that increases from 1.3% for post-KRR cohorts to 3.0% for those age 15 and over at the outset of the KRR (Table 2). Applying Likert-scale thresholds yield comparable estimates: 2.4% with the 17 original DSM symptoms only and fewer with all 21 HTQ symptoms in our survey (1.6%)—surprisingly to the extent the symptoms added in the HTQ list were intended to better capture the experience of Southeast Asian refugees. But two thirds of our respondents experience at least one of the PTSD symptoms (66.7%), a proportion that also increases steadily from the post-KRR cohorts (61.2%) to those age 15 and over at the outset of the KRR (70.2%). This contributes to prevalence estimates being sensitive to the types of symptoms that are required to assess probable PTSD. We thus find that 4.2% of our respondents meet the stringent criteria for partial PTSD, and 12.8% the lenient criteria for partial PTSD (Table 2). The inter-cohort pattern is the same with these two alternative definitions.

Next, our models of the likelihood of having experienced or witnessed traumatic events show strong cohort effects, as those born either during or after the KRR are consistently less likely to have experienced such events across event categories (Table 3). Most differences are significant, particularly for the lower likelihood of having experienced or witnessed deprivation and the unnatural death of a friend, or relative, or murder for post-KRR cohorts. Once these inter-cohort differences are accounted for, the only significant difference for respondents having lost a parent before age 20 concerns abuse, which bilateral orphans are significantly more likely to have experienced or witnessed than paternal (only) orphans. Male respondents are also significantly more likely to have experienced or witnessed life-threatening conditions.

Finally, our models of self-reported health status and symptoms show highly significant traumatic-exposure and cohort effects for self-rated health, anxiety, depression, and somatic symptoms, without significant differences between non-orphans and the different orphanhood categories once exposure to traumatic events and to the KRR are controlled for. (Results for depression shown in Table 4.)

With respect to PTSD, we find that meeting the DSM-4 criteria for full PTSD is significantly less common among non-orphans but also among bilateral orphans than among paternal- or maternal-only orphans (the difference between the last two is not significant, Table 4). With these criteria for plausible PTSD, the effects are significant across all models, whereas cohort effects no longer are. Results for the two alternative sets of criteria for partial PTSD, stringent and lenient, are similar but the coefficients for orphanhood types lose significance in some of the models. The same applies to results with alternative criteria for PTSD that are based on Likert-scale thresholds irrespective of symptom categories (results not shown).

## Discussion

Primarily concerned with the long-term consequences of parental loss on mental health, our study is situated in Cambodia. Due to the extreme levels of adult mortality during the KRR, we expect parental loss to have been more common and less selective in Cambodia than in most other settings. As extensively documented, the KRR not only led to extreme mortality levels, but also widespread exposure to violence, abuse, and other potentially traumatic events. Several studies have convincingly documented the high prevalence of PTSD among refugees who fled Cambodia in the early 1980s. Fewer studies have attempted to assess mental health in Cambodia. These studies suggest a prevalence of PTSD much lower than among refugees interviewed in Western countries but still higher than typical in general populations. One issue traversing this literature is whether the Western-based definition of and survey instruments for assessing probable PTSD aptly capture the impact on mental health of what the KRR survivors have endured.

In this paper, we first document the extremely high exposure to traumatic events in the Cambodian population. Nearly three in four respondents aged 20 and older report have directly experienced or witnessed at least one traumatic event (73.7%, Table 2). As expected, these experiences are more common among those born before the KRR, but consistent with the largest national study to date (Schunert et al. und.), we find that they are quite common even among post-KRR cohorts (71.7%). Once these cohort differences are accounted for, we find that bilateral orphans are more likely to have experienced or witnessed such events, significantly so in the case of abuse (Table 3).

A majority of our respondents also reports experiencing somatic symptoms (57.4%) and anxiety symptoms (56.0%), and a majority of those aged 15 and over at the outset of the KRR report feeling sad, or in pain or suffering (50.3%, compared to 37.7% for all respondents) and are probably depressed (54.7% compared to 42.8% for all respondents, Table 2). Using the DSM-4 criteria, the prevalence of full PTSD (2.3%) is also high compared to most populations around the world (Sack et al., 1996), albeit lower than in the national survey (2.7%, Schunert et al. und.). Our sample was not drawn to be nationally-representative, however, and our self-reports only included a subset of the symptoms list included in the HTQ (21 out of 40). Alternative criteria yielded prevalence estimates ranging from 1.6 to 12.8%, with two thirds of respondents reporting at least one of the 21 PTSD symptoms. Across conditions and definitions, we consistently observed with only minor exceptions higher prevalence among respondents who experienced the KRR, especially those who did so as adults. While this pattern was expected, the estimated prevalence of various mental health disorders was only slightly lower for post-KRR cohorts for most disorders, with the possible exception of depression.

Having established these differences in exposure to traumatic events and these inter-cohort differences, our analyses focused on the effects of losing one or both parents before age 20. We observed two different patterns. With respect to probable depression, inter-cohort effects are highly significant and orphanhood effects are not. With respect to probable PTSD, orphanhood effects are significant and inter-cohort effects no longer are. Our study is limited to KRR survivors who also survived to the time of the survey, in 2012 or 2014. Mortality selection (higher mortality rates for those suffering mental health disorders) may bias our estimates of the effect of a given characteristic if this mortality selection differs for individuals with and without that characteristic. Among the variables in our mental health models, this is most likely to be the case for being a member of earlier cohorts (i.e., older). This may cause coefficients to be biased towards zero and to lose significance for the earlier cohorts in our PTSD models.

The significant orphanhood effects suggest that both non-orphans and bilateral orphans are, relative to paternal- or maternal-only orphans, less likely to suffer from PTSD. The results for non-orphans may not seem surprising, yet the loss of a parent would only be captured by standard lists of traumatic events when resulting from unnatural deaths or murder—a condition that, considering these results, appears unnecessarily restrictive. Controlling for exposure to traumatic events as usually defined, we still find that the loss of a parent during childhood or adolescence increases the risk of subsequently experiencing PTSD, as consistent with extant studies of bereavement (Schaal et al., 2010; Morina et al., 2011; Heeke et al., 2017).

The results for bilateral orphans are more surprising: while more likely to experience trauma, especially abuse, bilateral orphans are less likely, given traumatic exposure, to exhibit PTSD symptoms later. We believe this indicates different post-traumatic trajectories for paternal or maternal orphans and for bilateral orphans. Children who lost one parent are more likely to remain in the same household as the surviving parent. Surviving parents may themselves experience grief and trauma, which could in turn impact their parenting of these orphans (Field et al., 2011). Intergenerational studies document a higher prevalence of PTSD among adolescents who have at least one parent with PTSD (Sack et al., 1995). Children who lost both of their parents are more likely to transition to a new household, most often headed by a relative (e.g., grandparents, uncles and aunts). The results for bilateral orphans are also consistent with the hypothesis of positive selection resulting from better-off households being more likely to take in these orphans. In any event, while we expected that, relative to paternal or maternal orphans, bilateral orphans might fare worse due to their double exposure to parental loss, these results would suggest rather that paternal or maternal orphans might be worse off from their double exposure to a parental loss and being cared for by a grieving parent.

Based on various critiques of applying the Western categories embodied in the DSM criteria for PTSD across cultural settings and our own difficulties in administering the full versions of the HSCL-25 and the HTQ during pilot testing, we initiated our data collection and analyses with a fair amount of skepticism regarding the applicability of these criteria and survey instruments in this population. We only administered a reduced version of the HSCL-25 and HTQ and modelled probable PTSD with various alternatives to the DSM-4 criteria. The estimates of PTSD prevalence varied across measures, most of them yielding higher estimates than the one based on DSM-4 criteria (by design in the case of “partial” PTSD criteria), but we do not have an externally validated prevalence estimate to adjudicate between those. In modelling the effects of having experienced traumatic events, the KRR regime, and parental loss, however, patterns were most consistent with the DSM-4 criteria for full PTSD. While this is not a formal test of the cross-cultural validity of the DSM definition, alternative measures appear to rest on more “noisy” criteria for probable PTSD. For this study of the factors contributing to probable PTSD, the measure based on the DSM-4 criteria did perform better than these alternatives.

## Figures and Tables

**Table 1:** Independent variables, descriptive statistics by birth cohorts, adults aged 20 and over

	1960 & earlier	1961 to 1974	1975 to 1978	1979 to 1988	All Cohorts	*N*
Orphanhood						
Non orphans	43.4%	32.3%	37.8%	39.9%	37.5%	1,598
Paternal	21.2%	37.6%	38.1%	35.3%	32.5%	1,385
Maternal	21.0%	13.8%	12.3%	17.0%	16.4%	697
Bilateral	14.4%	16.3%	11.8%	7.8%	13.6%	581
Gender						
Female	61.8%	56.8%	57.7%	57.7%	58.5%	2,492
Male	38.2%	43.2%	42.3%	42.3%	41.5%	1,769
Marital status						
Never married	3.9%	4.8%	9.2%	29.1%	9.9%	421
Currently married	81.6%	87.4%	84.3%	67.8%	81.5%	3,471
Separated/divorced	3.8%	3.3%	3.1%	2.5%	3.3%	140
Widowed	10.8%	4.5%	3.4%	60.0%	5.4%	229
Educational attainment						
Missing	3.5%	1.8%	2.0%	2.8%	2.5%	107
No formal schooling	23.1%	20.3%	20.4%	17.9%	20.6%	879
Some primary	51.0%	49.1%	47.6%	45.4%	48.8%	2,078
Complete primary only	14.4%	18.9%	23.0%	22.2%	18.7%	795
Complete lower secondary	8.0%	9.8%	7.0%	11.7%	9.4%	402
Region						
Urban	30.8%	30.2%	21.8%	29.8%	29.6%	1,260
Peri-urban	20.6%	23.8%	23.2%	29.9%	24.1%	1,026
Rural, close	35.0%	29.6%	31.4%	23.1%	29.9%	1,275
Rural, remote	13.7%	16.5%	23.5%	17.2%	16.4%	700
*N*	1,216	1,816	357	872	4,261	

**Table 2:** Traumatic events and symptoms of mental health disorder, prevalence of by birth cohorts, adults aged 20 and over (in percent)

	1960 & earlier	1961 to 1974	1975 to 1978	1979 to 1988	All cohorts	*N*
Trauma event						
Deprivation	64.5%	62.0%	51.9%	56.1%	60.7%	3,729
Life threatening	44.5%	47.3%	40.9%	44.1%	45.3%	4,090
Abuse	25.6%	25.5%	20.7%	21.7%	24.3%	4,165
Unnatural death, murder	16.7%	16.3%	11.8%	11.8%	15.1%	4,209
Any event	74.9%	75.1%	67.9%	71.7%	73.7%	3,775
Self-rated status						
Feeling pain, suffering or feeling sad	50.3%	38.2%	27.1%	23.6%	37.7%	4,186
HSCL measures						
Anxiety	60.3%	57.4%	47.5%	50.5%	56.0%	4,243
Depression	54.7%	44.0%	30.7%	28.8%	42.8%	4,126
Any somatic symptom	66.2%	58.0%	45.8%	47.4%	57.4%	3,561
HTQ measures						
Full PTSD (DSM-4 symptom typology)	3.0%	2.2%	2.2%	1.3%	2.3%	4,216
Stringent partial PTSD (typology)	5.3%	4.3%	3.4%	2.9%	4.2%	4,189
Lenient partial PTSD (typology)	15.0%	13.6%	11.9%	8.6%	12.8%	4,178
PTSD symptoms (17 original DSM-4)	3.2%	2.2%	2.2%	1.5%	2.4%	4,214
PTSD symptoms (21 of HTQ 40)	2.1%	1.6%	2.0%	1.0%	1.6%	4,220
Any symptom (21 of HTQ 40)	70.2%	67.3%	64.8%	61.2%	66.7%	3,605

**Table 3:** Traumatic events, likelihood of having directly experienced or witnessed, adults aged 20 and over (standardized *β* coefficients; *p*-values in parentheses)

	Deprivation	Life threatening situation	Abuse	Unnatural death (friend/relative), or murder	Any
Orphanhood (ref = Paternal Orphan)					
Not Orphaned	0.028	−0.054	0.027	−0.089	−0.059
	(0.737)	(0.470)	(0.792)	(0.546)	(0.553)
Maternal Orphan	0.120	−0.002	0.155	0.173	0.077
	(0.134)	(0.974)	(0.101)	(0.203)	(0.425)
Bilateral Orphan	0.078	0.131	0.231*	0.177	0.191
	(0.327)	(0.065)	(0.012)	(0.172)	(0.057)
Male	0.042	0.268***	0.043	−0.082	0.165
	(0.557)	(0.000)	(0.612)	(0.507)	(0.057)
Birth cohorts (ref = 1961 to 1974)					
1960 and earlier	0.088	−0.059	0.004	0.055	−0.005
	(0.264)	(0.405)	(0.962)	(0.674)	(0.961)
1975 to 1978	−0.172*	−0.150*	−0.193*	−0.227	−0.159
	(0.017)	(0.027)	(0.037)	(0.103)	(0.064)
1979 or later	−0.208**	−0.099	−0.180	−0.479***	−0.145
	(0.006)	(0.155)	(0.056)	(0.001)	(0.118)
Geographic area (ref = Peri-urban)					
Urban	0.904***	0.102	−0.226*	−0.534***	1.130***
	(0.000)	(0.200)	(0.037)	(0.000)	(0.000)
Rural, closest	−0.058	−0.198*	0.025	−1.179***	−0.042
	(0.495)	(0.014)	(0.812)	(0.000)	(0.676)
Rural, furthest	−0.160*	0.303***	0.241*	−1.624***	0.010
	(0.046)	(0.000)	(0.012)	(0.000)	(0.914)
Model statistics					
Observations	3,729	4,090	4,165	4,209	3,775
Pseudo R2	0.041	0.013	0.008	0.042	0.038

Notes ^12^

(Prob. > χ^2^) < 0.0001 for all models.

* Significant at the 5% level;

** Significant at the 1% level;

*** Significant at the 0.1% level

**Table 4:** Probable depression and post-traumatic stress disorder, likelihood of experiencing at the time of survey, adults aged 20 and over (standardized *β* coefficients; *p*-values in parentheses)

	Probable Depression				Probable PTSD (DSM-4 criteria)			
	Model I	Model II	Model III	Model IV	Model I	Model II	Model III	Model IV
Orphanhood (ref = Paternal Orphan)								
Not Orphaned	−0.033	−0.010	−0.028	−0.013	−1.800*	−1.884*	−1.860*	−1.882*
	(0.679)	(0.907)	(0.740)	(0.872)	(0.033)	(0.026)	(0.027)	(0.027)
Maternal orphan	−0.004	0.008	−0.001	−0.004	−0.058	−0.124	−0.210	−0.214
	(0.955)	(0.923)	(0.987)	(0.956)	(0.934)	(0.860)	(0.765)	(0.762)
Bilateral orphan	0.063	0.052	0.038	0.050	−3.109**	−3.662**	−3.740**	−3.670**
	(0.397)	(0.509)	(0.630)	(0.522)	(0.005)	(0.003)	(0.002)	(0.002)
Birth cohorts (ref = 1961 to 1974)								
1960 and earlier	0.355***	0.333***	0.336***	0.346***	0.670	0.736	0.748	0.774
	(0.000)	(0.000)	(0.000)	(0.000)	(0.371)	(0.329)	(0.320)	(0.306)
1975 to 1978	−0.302***	−0.273***	−0.261***	−0.266***	0.119	0.340	0.428	0.437
	(0.000)	(0.000)	(0.001)	(0.001)	(0.874)	(0.645)	(0.563)	(0.556)
1979 or later	−0.522***	−0.452***	−0.450***	−0.429***	−1.750	−1.571	−1.475	−1.318
	(0.000)	(0.000)	(0.000)	(0.000)	(0.072)	(0.108)	(0.131)	(0.180)
Any traumatic experience		0.550***	0.332***	0.472***		3.244**	2.537*	2.388*
		(0.000)	(0.000)	(0.000)		(0.001)	(0.016)	(0.021)
Abuse			0.560***				1.642*	
			(0.000)				(0.018)	
Unnatural death, murder				0.294***				2.200***
				(0.000)				(0.000)
Male	−0.468***	−0.475***	−0.483***	−0.469***	−0.732	−0.997	−1.008	−0.901
	(0.000)	(0.000)	(0.000)	(0.000)	(0.362)	(0.216)	(0.210)	(0.264)
Marital status (ref = Currently married)								
Never married	−0.107	−0.103	−0.095	−0.104	0.533	0.409	0.437	0.423
	(0.154)	(0.186)	(0.225)	(0.184)	(0.479)	(0.591)	(0.566)	(0.581)
Separated/Divorced	−0.026	0.005	0.020	0.008	−0.308	−0.619	−0.596	−0.527
	(0.693)	(0.942)	(0.780)	(0.915)	(0.672)	(0.466)	(0.483)	(0.532)
Widowed	0.054	0.050	0.057	0.045	−1.090	−1.696	−1.693	−1.669
	(0.425)	(0.485)	(0.436)	(0.537)	(0.229)	(0.115)	(0.115)	(0.121)
Education (ref = Incomplete primary)								
No formal schooling	0.025	0.019	−0.003	0.021	1.042	1.025	1.006	1.176
	(0.722)	(0.803)	(0.969)	(0.782)	(0.115)	(0.127)	(0.133)	(0.081)
Complete primary only	−0.176*	−0.220**	−0.220**	−0.231**	−3.529**	−3.294**	−3.271**	−3.332**
	(0.016)	(0.004)	(0.005)	(0.003)	(0.005)	(0.008)	(0.008)	(0.007)
Complete secondary	−0.370***	−0.396***	−0.397***	−0.396***	−1.549	−1.390	−1.374	−1.430
	(0.000)	(0.000)	(0.000)	(0.000)	(0.147)	(0.179)	(0.182)	(0.166)
Geographic area (ref = Peri-urban)								
Urban	−0.129	−0.161	−0.095	−0.111	−1.083	−1.248	−1.008	−0.703
	(0.137)	(0.083)	(0.309)	(0.235)	(0.231)	(0.169)	(0.266)	(0.446)
Rural, closest	−0.310***	−0.201*	−0.226*	−0.149	−0.725	−0.408	−0.417	0.104
	(0.000)	(0.023)	(0.011)	(0.097)	(0.359)	(0.608)	(0.599)	(0.898)
Rural, furthest	−0.442***	−0.398***	−0.442***	−0.345***	−5.197***	−4.854**	−4.920**	−4.182**
	(0.000)	(0.000)	(0.000)	(0.000)	(0.001)	(0.001)	(0.001)	(0.006)
Model statistics Observations	4022	3581	3547	3559	4,110	3,655	3,619	3,632
Pseudo R2	0.054	0.062	0.073	0.065	0.073	0.095	0.103	0.112

Notes ^12^

(Prob. > χ^2^) < 0.0001 for all models.

* Significant at the 5% level;

** Significant at the 1% level;

*** Significant at the 0.1% level

## References

[R1] Bicego G, Rutstein S, & Johnson K (2003). Dimensions of the emerging orphan crisis in sub-saharan Africa. Social Science & Medicine, 56(6), 1235–1247. 10.1016/S0277-9536(02)00125-9 12600361

[R2] Carlson EB, & Rosser-Hogan R (1991). Trauma experiences, posttraumatic stress, dissociation and depression in Cambodian refugees. American Journal of Psychiatry, 148(11), 1548–1551. 10.1176/ajp.148.11.1548 1928471

[R3] Carlson EB, & Rosser-Hogan R (1993). Mental health status of Cambodian refugees ten years after leaving their homes. American Journal of Orthopsychiatry, 63(2), 223–231. 10.1037/h0079422 8484428

[R4] Carlson EB, & Rosser-Hogan R (1994). Cross-cultural response to trauma: A study of traumatic experiences and posttraumatic symptoms in Cambodian refugees. Journal of Traumatic Stress, 7(1), 43–58. 10.1007/BF02111911 8044442

[R5] Case A, Paxson C, & Ableidinger J (2004). Orphans in Africa: Parental death, poverty, and school enrollment. Demography, 41(3), 483–508. 10.1353/dem.2004.0019 15461011

[R6] Chhim S (2013). *Baksbat* (broken courage): A trauma-based cultural syndrome in Cambodia. Medical Anthropology, 32(2), 160–173. 10.1080/01459740.2012.674078 23406066

[R7] Cluver L, & Orkin M (2009). Cumulative risk and Aids-orphanhood: Interactions of stigma, bullying and poverty on child mental health in South Africa. Social Science & Medicine, 69(8), 1186–1193. 10.1016/j.socscimed.2009.07.033 19713022

[R8] de Jong JT, Komproe IH, Van Ommeren M, Masri E, Araya M, Khaled M, van De Put N,W, and, & Somsundaram D (2001). Lifetime events and posttraumatic stress disorder in 4 post-conflict settings. Journal of the American Medical Association, 286(5), 555–562. 10.1001/jama.286.5.555 11476657

[R9] Dickstein BD, Weathers FW, Angkaw AC, Nievergelt CM, Yurgil K, Nash WP, Baker DG, Litz BT, & the Marine Resiliency Study Team. (2015). Diagnostic utility of the posttraumatic stress disorder (PTSD) checklist for identifying full and partial PTSD in active-duty military. Assessment, 22(3), 289–297. 10.1177/1073191114548683 25178804

[R10] Dubois V, Tonglet R, Hoyois P, Ka S, Rousseaux JP, & Hauff E (2004). Household survey of psychiatric morbidity in Cambodia. International Journal of Social Psychiatry, 50(2), 174–185. 10.1177/0020764004043125 15293434

[R11] Eisenbruch M (1991). From posttraumatic stress disorder to cultural bereavement: Diagnosis of southeast Asian refugees. Social Science and Medicine, 33(6), 673–680. 10.1016/0277-9536(91)90021-4 1957187

[R12] Field N, Om C, Kim T, & Vorn S (2011). Parental styles in second generation effects of genocide stemming from the Khmer Rouge regime in Cambodia. Attachment & Human Development, 13(6), 611–628. 10.1080/14616734.2011.609015 22011103

[R13] Hasanović M, Sinanović O, Selimbašić Z, Pajević I, & Avdibegović E (2006). Psychological disturbances of war-traumatized children from different foster and family settings in Bosnia and Herzegovina. Croatian Medical Journal, 47(1), 85–94. https://www.ncbi.nlm.nih.gov/pmc/articles/PMC2080380/pdf/CroatMedJ_47_0085.pdf 16489701 PMC2080380

[R14] Heeke C, Stammel N, Heinrich M, & Knaevelsrud C (2017). Conflict-related trauma and bereavement: Exploring differential symptom profiles of prolonged grief and posttraumatic stress disorder. Bmc Psychiatry, 17(1), 118. 10.1186/s12888-017-1286-2 28356093 PMC5372336

[R15] Heuveline P (2001). Approaches to measuring genocide: Excess mortality during the Khmer Rouge period. In Chirot D, & Seligman M (Eds.), Ethnopolitical Warfare: Causes, consequences, and possible solutions (pp. 93–108). American Psychological Association.

[R16] Heuveline P (2015). The boundaries of genocide: Quantifying the uncertainty of the death toll during the Pol Pot regime in Cambodia (1975–79). Population Studies, 69(2), 201–218. 10.1080/00324728.2015.1045546 26218856 PMC4562795

[R17] Heuveline P, & Kao Nakphong M (2023). Contemporary marriage in Cambodia. Journal of Family Issues, 0(0). 10.1177/0192513X231155590 PMC11244510 39005494

[R18] Hinton DE, Pich V, Marques L, Nickerson A, & Pollack MH (2010). Khyâl attacks: A key idiom of distress among traumatized Cambodia refugees. Culture Medicine & Psychiatry, 34(2), 244–278. 10.1007/s11013-010-9174-y 20407813

[R19] Hinton D, Hinton S, Um K, Chea A, & Sak S (2002). The Khmer weak heart syndrome: Fear of death from palpitations. Transcultural Psychiatry, 39(3), 323–344. 10.1177/136346150203900303 20814562 PMC2931422

[R20] Inter-university Consortium for Political and Social Research, & distributor. (2017). The Mekong Island Population Laboratory (MIPopLab), a demographic Surveillance System in Rural Cambodia (2000–06). ICPSR36601-v1. 10.3886/ICPSR36601.v1

[R21] Jones TM, Nurius P, Song C, & Fleming CM (2018). Modeling life course pathways from adverse childhood experiences to adult mental health. Child Abuse & Neglect, 80(2), 32–40. 10.1016/j.chiabu.2018.03.005 29567455 PMC5953821

[R22] Kristensen P, Weisæth L, & Heir T (2012). Bereavement and mental health after sudden and violent losses: A review. Psychiatry, 75(1), 76–97. 10.1521/psyc.2012.75.1.76 22397543

[R23] Marshall GN, Schell TL, Elliott MN, Berthold SM, & Chun CA (2005). Mental health of Cambodian refugees 2 decades after resettlement in the United States. The Journal of the American Medical Association, 294(5), 571–579. 10.1001/jama.294.5.571 16077051

[R24] Mollica RF, Caspi-Yavin Y, & Bollini P (1992). The Harvard Trauma Questionnaire: Validating a cross-cultural instrument for measuring torture, trauma, and posttraumatic stress disorder in indochinese refugees. Journal of Nervous and Mental Disease, 180(2), 111–116. 10.1097/00005053-199202000-00008 1737972

[R25] Mollica RF, Wyshkah G, de Marnette G, & Khoun D (1987). Indochinese versions of the Hopkins Symptom Checklist – 25: A screening instrument for the psychiatric case of refugees. American Journal of Psychiatry, 144(4), 497–500. 10.1176/ajp.144.4.497 3565621

[R26] Morina N, von Lersner U, & Prigerson HG (2011). War and bereavement: Consequences for mental and physical distress. PLOS ONE, 6(7), e22140. 10.1371/journal.pone.0022140 21765944 PMC3134481

[R27] Ong K, Cherng I, Yi S, Tuot S, Chhoun P, Shibanuma A, Yasuoka J, & Jimba M (2015). What are the factors associated with depressive symptoms among orphans and vulnerable children in Cambodia? BMC Psychiatry, 15(1), 178. 10.1186/s12888-015-0576-9 26220677 PMC4517410

[R28] Palmieri PA, Marshall GN, & Schell TL (2007). Confirmatory factor analysis of posttraumatic stress symptoms in Cambodian refugees. Journal of Traumatic Stress, 20(2), 207–216. 10.1002/jts.20196 17427910

[R29] Puffer ES, Drabkin AS, Stashko AL, Broverman SA, Ogwang-Odhiambo RA, & Sikkema KJ (2012). Orphan status, Hiv risk behavior, and mental health among adolescents in rural Kenya. Journal of Pediatric Psychology, 37(8), 868–878. 10.1093/jpepsy/jss077 22728899 PMC3437686

[R30] Sack WH, Clarke GN, & Seeley J (1995). Posttraumatic stress disorder across two generations of Cambodian refugees. Journal of the American Academy of Child & Adolescent Psychology, 34(9), 1160–1166. 10.1097/00004583-199509000-00013 7559310

[R31] Sack WH, Clarke GN, & Seeley J (1996). Multiple forms of stress in Cambodian adolescent refugees. Child Development, 67(1), 107–116. 10.2307/1131689 8605822

[R32] Sack WH, Seeley J, & Clarke GN (1997). Does PTSD transcend cultural barriers? A study from the Khmer Adolescent Refugee Project. Journal of the American Academy of Child & Adolescent Psychiatry, 36(1), 49–54. 10.1097/00004583-199701000-00017 9000781

[R33] Schaal S, Jacob N, Dusingizemungu JP, & Elbert T (2010). Rates and risks for prolonged grief disorder in a sample of orphaned and widowed genocide survivors. Bmc Psychiatry, 10(1), 55. 10.1186/1471-244X-10-55 20604936 PMC2914709

[R34] Schaal S, Weierstall R, Dusingizemungu JP, & Elbert T (2012). Mental health 15 years after the killings in Rwanda: Imprisoned perpetrators of the genocide against the Tutsi versus a community sample of survivors. Journal of Traumatic Stress, 25(4), 446–453. 10.1002/jts.21728 22865747

[R35] Schunert T, Khann S, Kao S, Pot C, Saupe LB, Sek S, & Nhong H (undated) (Eds.). Cambodian Mental Health Survey 2012. Department of Psychology, Royal University of Phnom Penh.

[R36] Sliwinski M (1995). Le génocide Khmer Rouge: Une analyse démographique [The Khmer Rouge Genocide: A demographic analysis]. L’Harmattan.

[R37] Sonis J, Gibson JL, de Jong JTV, Field NP, Hean S, & Komproe I (2009). Probable post-traumatic stress disorder and disability in Cambodia: Associations with perceived justice, desire for revenge, and attitudes toward the Khmer Rouge trials. The Journal of the American Medical Association, 302(5), 527–536. 10.1001/jama.2009.1085 19654387

[R38] Steel Z, Chey T, Silove D, Marnane C, Bryant RA, & van Ommeren M (2009). Association of torture and other potentially traumatic events with mental health outcomes among populations exposed to mass conflict and displacement: A systematic review and meta-analysis. The Journal of the American Medical Association, 302(5), 537–549. 10.1001/jama.2009.1132 19654388

[R39] Wilkins KC, Lang AJ, & Norman SB (2011). Synthesis of the psychometric properties of the PTSD checklist (PCL) military, civilian, and specific versions. Depression and Anxiety, 28(7), 596–606. 10.1002/da.20837 21681864 PMC3128669

